# Loss of liver kinase B1 in human seminoma

**DOI:** 10.3389/fonc.2023.1081110

**Published:** 2023-03-10

**Authors:** Manish Kumar, Subhransu S. Sahoo, M. Fairuz B. Jamaluddin, Pradeep S. Tanwar

**Affiliations:** School of Biomedical Sciences and Pharmacy, University of Newcastle, Callaghan, NSW, Australia

**Keywords:** germ cells, mTOR, TCam-2, testicular cancer, serine/threonine kinase 11

## Abstract

Testicular cancer is a common malignancy of young males and is believed to be originated from defective embryonic or adult germ cells. Liver kinase B1 (LKB1) is a serine/threonine kinase and a tumor suppressor gene. LKB1 is a negative regulator of the mammalian target of rapamycin (mTOR) pathway, often inactivated in many human cancer types. In this study, we investigated the involvement of LKB1 in the pathogenesis of testicular germ cell cancer. We performed immunodetection of LKB1 protein in human seminoma samples. A 3D culture model of human seminoma was developed from TCam-2 cells, and two mTOR inhibitors were tested for their efficacy against these cancer cells. Western blot and mTOR protein arrays were used to show that these inhibitors specifically target the mTOR pathway. Examination of LKB1 showed reduced expression in germ cell neoplasia *in situ* lesions and seminoma compared to adjacent normal-appearing seminiferous tubules where the expression of this protein was present in the majority of germ cell types. We developed a 3D culture model of seminoma using TCam-2 cells, which also showed reduced levels of LKB1 protein. Treatment of TCam-2 cells in 3D with two well-known mTOR inhibitors resulted in reduced proliferation and survival of TCam-2 cells. Overall, our results support that downregulation or loss of LKB1 marks the early stages of the pathogenesis of seminoma, and the suppression of downstream signaling to LKB1 might be an effective therapeutic strategy against this cancer type.

## Introduction

Testicular cancer (TC) is the most common malignancy in young men, with an increasing incidence over the past 30 years (1, 2). It affects males during the peak of reproductive life and causes a decline in male reproductive health which is associated with reduced semen quality, increased germ cell abnormalities, and infertility (1, 3). As per the World Health Organization (WHO) classification, TC is divided into three main types: germ cell tumors (GCTs), sex cord stromal tumors, and mixed germ cell sex cord stromal tumors. GCTs are further subdivided into two major subtypes, germ cell neoplasia *in situ* (GCNIS)-derived and not GCNIS-derived (4). GCNIS-derived tumors are categorized into five different types, including seminoma, trophoblastic tumors (choriocarcinoma and other trophoblastic tumors), embryonal carcinoma, teratoma (post-pubertal type) and yolk sac tumors (4). GCTs are the most common (>95% of cases) form of TC (5). Among GCTs, seminoma is the most common type and accounts for approximately 50% of cases (6).

GCTs originate from the uncontrolled division of germ cells that fail to mature and differentiate (7). These cancer cells are believed to arise from primordial germ cells either during their migration to genital ridges or mitotic division to increase germ cell numbers after they reach genital ridges (8). TC initiates as GCNIS, a pre-invasive cancer stage in which the cancer cells express primordial germ cell markers. During the onset of puberty, GCNIS cells proliferate and develop neoplastic characteristics leading to TC (9). TCs are successfully treated by orchidectomy followed by cisplatin-based chemotherapy, however, treatment reduces patient fertility. Moreover, 20-30% of TC patients do not respond to standard chemotherapy or relapse (10, 11). Only 20-25% of these unresponsive patients respond to salvage chemotherapy in combination with cisplatin and previously unused drugs (11, 12). Non-germinomatous germ cell tumors, a form of GCTs which may be located in the central nervous system, have a poor prognosis with less than 10% 5-year survival rate (13). The reasons for this poor prognosis are unknown molecular mechanisms underlying the development of both chemotherapy-sensitive and resistant tumors. Previous efforts to treat these unresponsive cases of TC have mostly led to disappointing results due to a lack of full understanding of mechanisms underlying the resistance development (14). Thus, identifying the mechanisms involved in the initiation of GCNIS, the progression of GCNIS into TC, and the development of chemotherapy-resistant tumors will potentially facilitate early diagnosis, improved prognosis, and less invasive therapeutic approaches, especially in case of relapse/unresponsive cancer.

mTOR pathway is a major regulator of cell growth and division, and activating mutations have been reported in many different cancer types, including TC (13, 15–17). Balanced mTOR signaling is essential for the proliferation and differentiation of spermatogonial cells and the male fertility (18). LKB1 is a negative regulator of the mTOR pathway, and its inactivation in patients and mice predispose them to early onset of tumors (19). Moreover, loss of LKB1 in Peutz-Jeghers syndrome (PJS) is well-known to cause large-cell calcifying Sertoli cell tumors of the testes (LCCSCTs) (20). This led us to investigate the role of LKB1 in human GCTs. In this study, we showed downregulation of LKB1 expression in seminoma patients and suppression of the mTOR pathway in a seminoma cell line resulted in reduced cell viability and proliferation.

## Materials and methods

### Human and mouse testis tissue samples

The use of human TC tissue samples in this study was approved by the Institutional Human Research Ethics Committee at The University of Newcastle, NSW, Australia. Human GCTs tissue samples (n = 13) were obtained from Hunter Cancer Biobank. High-density tumor microarrays (TMA) were obtained from Biomax (US Biomax, MD, USA). It included 48 tissue cores (35 seminoma, 5 non-seminoma and 8 normal). For quality control of TMA at Biomax, each single tissue spot on every array slide is individually examined by pathologists and certified according to WHO-published standardizations for the diagnosis and classification of TC. All mouse experiments performed were approved by the Animal Care and Ethics Committee of the University of Newcastle, Australia. Mouse care and experimental protocols were performed strictly under New South Wales Animal Research Act, New South Wales Animal Research Regulation, and the Australian code for the care and use of animals for scientific purposes guidelines. Testes from three adult C57BL/6;129SvEv mixed genetic background mice were collected and processed for histopathology as described (21).

### Cell culture

TCam‐2 cell line was kindly provided by Prof. Eileen McLaughlin (University of Newcastle). These cells were cultured in RPMI1640 (Gibco, Invitrogen, CA, USA), with penicillin/streptomycin (Gibco) and 10% fetal bovine serum (FBS, Bovogen Biologicals Pty, Vic, Australia) in an incubator with 5% CO_2_ and 37°C temperature (22). For 3D culture, TCam-2 cells were cultured on 96-well culture plates double-coated with polyHEMA [poly(2-hydroxyethyl methacrylate); Sigma-Aldrich, MO, USA]. PolyHEMA solution was prepared by dissolving 1.5 g PolyHEMA in 5 ml molecular biology grade water and 95 ml absolute ethanol (Sigma-Aldrich) at 65°C for 6 hours (23, 24). 5000 cells were cultured in each well for seven days with media change every 2 days. The images were taken with JuLi^TM^ Stage Real-Time Cell History Recorder (Nanoentek, Seoul, South Korea) in a humidified incubator with 5% CO_2_ and 37˚C temperature. For carboplatin, BEZ235, and everolimus treatments, 25000 cells in 50 µl Matrigel (Invitrogen, CA, USA) were plated in 24 well plates and cultured for seven days with media change every two days. For colony formation efficiency and colony death, the number of colonies were manually counted in each well. The viability of the cells cultured in the presence of control (DMSO), BEZ235, everolimus (dissolved in DMSO) and cisplatin plus BEZ235/everolimus was assessed using Vision Blue Cell Viability Fluorometric Assay (BioVision, CA, USA) performed as per manufacturer’s instructions (25). Each treatment was done in triplicate and every experiment was repeated three times.

### Immunohistochemistry (IHC) and immunofluorescence (IF)

IHC was performed as described in (21). Briefly, after deparaffinization and rehydration of paraffin sections following standard procedures, heat induced epitope retrieval was carried out in sodium citrate antigen unmasking buffer (pH 6.0) using a decloaking chamber (Biocare Medical, CA, USA) at 110˚C for 20 min. After inactivation of endogenous peroxidases with 0.3% H_2_O_2_ in absolute methanol and blocking with 10% goat serum in phosphate buffer saline, primary antibody (LKB1, 1:100 dilution, Plzf, 1:100 dilution, Santa Cruz Biotechnology, CA, USA; mTOR and p4EBP1, 1:100, Cell Signaling Technology, MA, USA; Stra8, 1:100 dilution, Abcam, VIC, Australia) was applied to the sections. Following incubation in HRP-tagged secondary goat anti-rabbit antibodies (Jackson ImmunoResearch Laboratories, PA, USA), the color was developed with DAB (3,3’-diaminobenzidine, Sigma-Aldrich) (21). For immunofluorescence, fluorophore tagged secondary antibodies (Jackson ImmunoResearch Laboratories, PA, USA) were used (26).

### Western blotting and mTOR array

TCam-2 cells were cultured in 6-well plates and were treated with BEZ235 (0-100 nmol/L) and everolimus (0-10 nmol/L) for 72 hours. Protein was extracted using RIPA buffer and equal amounts (27µg) of proteins were loaded and separated using sodium dodecyl sulfate-polyacrylamide gel electrophoresis (27). Following the transfer of proteins on a nitrocellulose membrane, they were incubated overnight at 4˚C in primary antibodies: pS6, S6 (1:1000, Cell Signaling Technology) and β-actin (1:2000, Sigma-Aldrich). HRP conjugated secondary antibodies (Jackson ImmunoResearch Laboratories) were used to detect the primary antibodies. The mean density of the protein bands was determined using NIH ImageJ plugin. β-actin was used as a loading control. Chemiluminescent mTOR Signaling Antibody Array was performed as per manufacturer’s instructions (Cell Signaling Technology). Equal amount of protein (27 µg) was loaded in each group. The array target map was obtained from the manufacturer’s homepage.

### Statistical analysis

All the statistical analysis was performed using Graph Pad Prism 6.0. The values are presented as mean ± SD. Student t-test was performed to calculate *P-*values. *P*-values less than 0.05 were considered statistically significant.

## Results

### LKB1 expression was downregulated in testicular cancer

**Figure 1:**
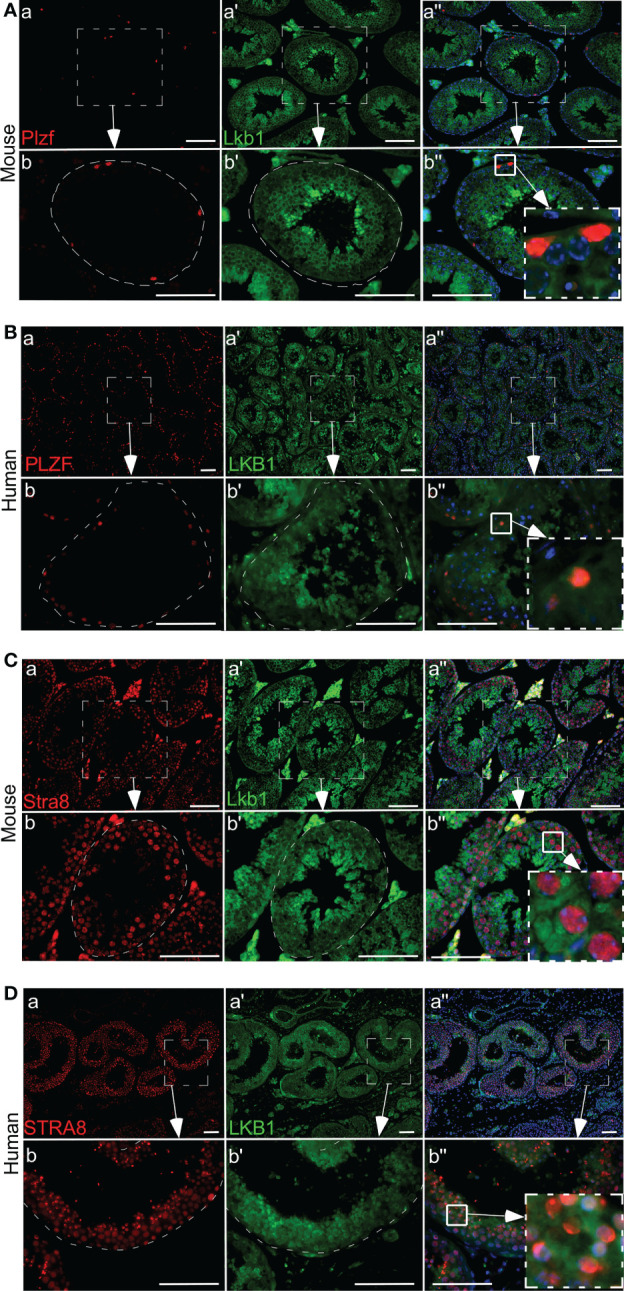
Differential expression of LKB1 in germ cells of human and mouse testis. **(A, B)** A low level of LKB1 expression is present in PLZF-positive spermatogonia in normal human and mouse testis. **(C, D)** Higher LKB1 protein expression is present in differentiated germ cells (STRA8). Areas outlined with squares in Ab’’, Bb”, CB”, and DB” are presented at higher magnification in insets. Bars: 100 µm.

**Figure 2:**
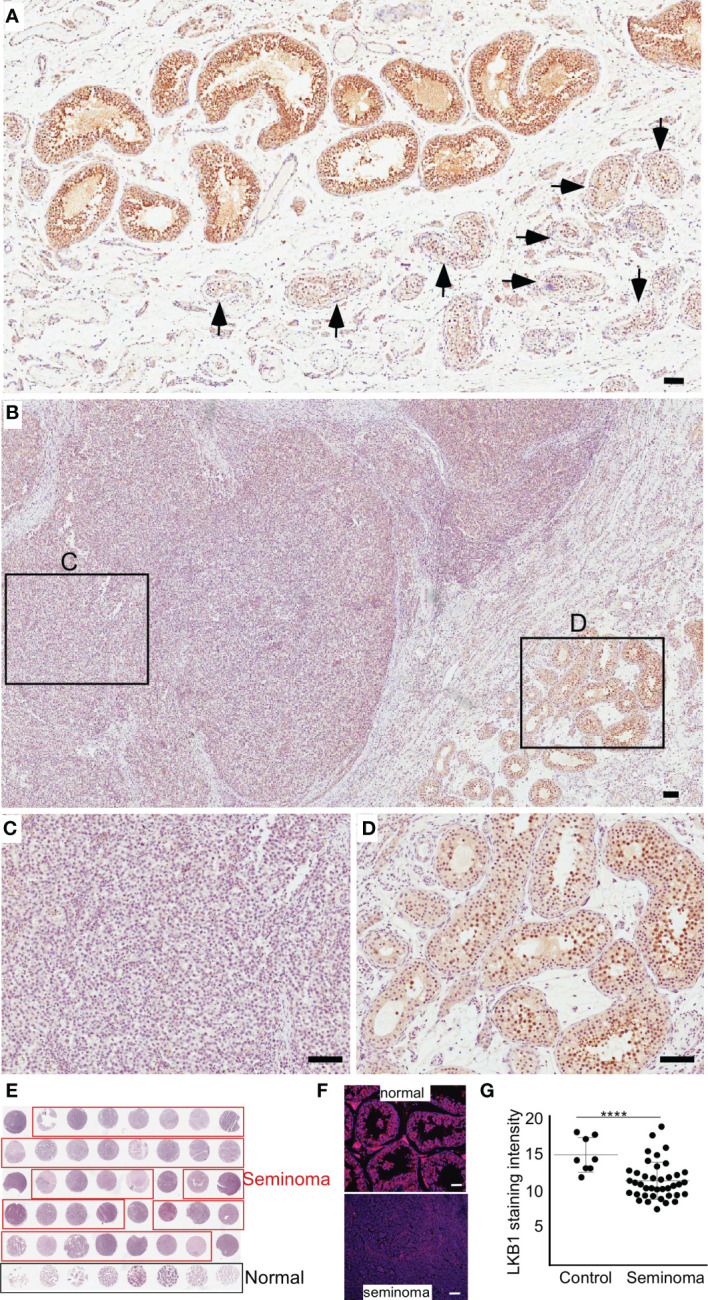
The loss of LKB1 expression is a common feature of seminoma. **(A)** LKB1 expression is present in normal-looking seminiferous tubules but absent in tubules with germ cell neoplasia *in situ* (arrows). **(B–D)** LKB1 protein expression is absent in cancerous germ cells but present in adjacent normal seminiferous tubules. **(E)** A representative image of testicular tissue array containing representative tissue samples from seminoma (n=35; outlined in red) and healthy controls (n=8; outlined in black). **(F)** Examples of LKB1 staining in tissue arrays cores representing seminoma and normal controls. **(G)** Quantification of LKB1 staining intensity between healthy controls and seminoma tissue samples. Bars: 100 µm.

Previously, we have established that *LKB1* is a tumor suppressor gene and a negative regulator of the mTOR pathway (28). In this study, we investigated the expression of LKB1 among human seminoma patient samples. In both human and mouse testis, LKB1 expression was higher in germ cells committed for differentiation cells (Stimulated by Retinoic Acid 8: STRA8+) than in spermatogonia (PLZF+, **Figure 1**; n=3/each). Compared to normal adjacent tubules, LKB1 expression was reduced in adjacent GCNIS lesions in the same patient (**Figure 2A**; n=5). Similarly, LKB1 expression was decreased or lost in seminoma, but intact LKB1 expression was present in adjacent normal-looking tubules in the same patient (**Figures 2B–D**). To further confirm the expression of LKB1 in a larger cohort of samples, we used a testicular cancer tissue array with 48 tissue cores (35 seminoma, 5 non-seminoma, and 8 normal; **Figure 2E**). In these additional patients, we also found a significant reduction in the expression of LKB1 protein in seminoma compared to controls (**Figures 2F, G**; *P* = 0.0007).

### Development of a 3D culture model of seminoma using TCam-2 cells

**Figure 3:**
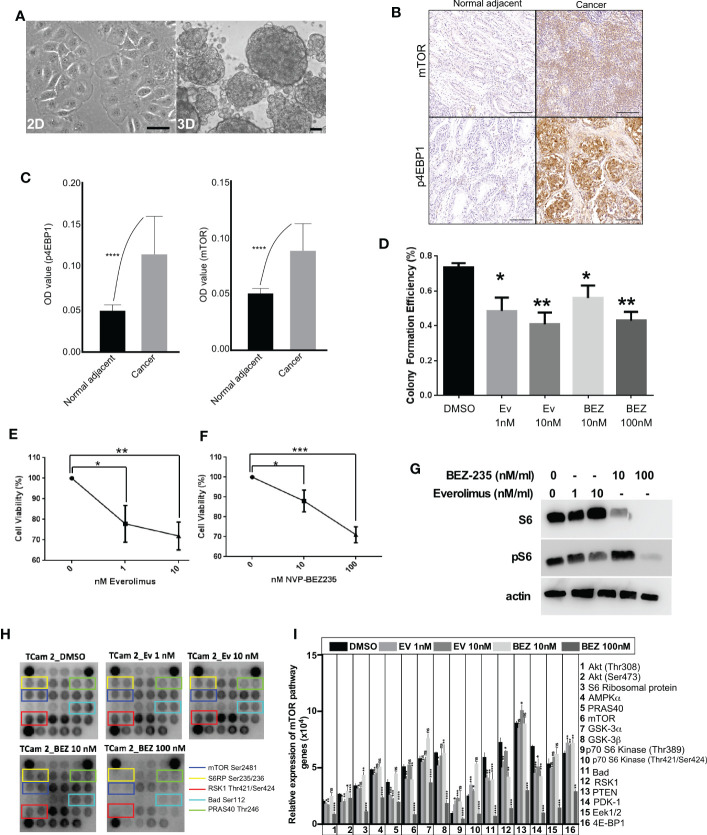
mTOR suppression inhibits the growth of human seminoma cells cultured in 3D. **(A)** Phase-contrast images of TCam-2 cells in 2D and 3D. **(B, C)** Increased mTOR and p4EBP1 expression in cancer tissues compared to normal adjacent. **(D)** TCam-2 cells were cultured (5 x 10^3^ cells/well) in 3D in a 96-well tissue culture plate. On Day 2, cells were treated with increasing concentrations (1-10 nM) of everolimus and (0-100 nM) of BEZ235. The efficiency of colony formation for TCam-2 cells were calculated in different drug concentrations (Number of colonies formed/total number of cells seeded, n=4 experiments). **(E, F)** Cell viability of TCam-2 cell line was assessed by VisionBlue™ fluorometric assay after treatment with increasing concentrations (0-10 nM) of everolimus and (0-100 nM) of BEZ235 (n = 3, *P < 0.05; **P < 0.01; ***P < 0.001). **(G)** Western blot depicting no change in the expression of S6 with everolimus treatment. However, BEZ235 treatment reduced S6 expression in TCam-2 cells. A dose-dependent decrease in pS6 expression was observed in both treatments. β-actin levels are shown for loading control. **(H)** mTOR protein array revealed a decrease in the activity levels of some of the key components of mTOR signaling after treatment with everolimus or BEZ235. **(I)** Bar diagram showing quantification of the differentially regulated proteins in mTOR array. Bar: 100um.

Two-dimensional (2D) cell culture system is a conventional *in vitro* model to study cellular responses to stimulations from biochemical and/or biophysical cues. Although 2D culture approaches have advanced our understanding of cancer cell growth, the results from 2D systems deviate significantly from *in vivo* response (29). For instance, some essential characteristics of cancer cells cannot be appropriately modeled in 2D cultures (30). To overcome this limitation, novel 3D cell culture platforms are being created that better mimic *in vivo* conditions (31–33). Previous studies on 3D culture have demonstrated that the presence of extracellular matrix (ECM) around cells significantly impacts cell proliferation, differentiation, and survival (34). Therefore, to mimic the 3D arrangement of cancer cells observed in GCTs and *in vivo* conditions, we cultured the TCam-2 cells as 3D spheroids. In 2D culture, TCam-2 cells showed flat morphology (**Figure 3A**), while in 3D culture, these cells grew in the form of spheroids where cells were connected in three dimensions (**Figure 3A**).

### Suppression of PI3K-mTOR signaling axis in seminoma cells after treatment with everolimus and BEZ235

In a previous study, we showed that the loss of the *LKB1* gene in the uterus leads to endometrial cancer development through hyperactive mTOR signaling and suppression of mTOR signaling in mice suppressed endometrial cancer growth (28). Due to the loss or reduced expression of LKB1 in seminoma samples, we decided to investigate if suppression of mTOR signaling in seminoma cells will affect their growth. First, we confirmed that mTOR signaling is upregulated in seminoma by examining the expression of mTOR and p4EBP1 (**Figures 3B, C**). To downregulate the mTOR pathway, we used two FDA-approved mTOR inhibitors (everolimus and BEZ235). We cultured the TCam-2 cells in 3D in the presence of different concentrations of everolimus (1 and 10 nM) and BEZ235 (10 and 100 nM) and found a reduction in colony number among all the concentrations used for both the drugs (**Figure 3D**). To investigate the reasons for reduced colony number, we examined the viability of these cells. We found a progressive and significant decline in the viability of TCam-2 cells cultured in the presence of increasing concentrations of everolimus and BEZ235 (**Figures 3E, F**).

To confirm the downregulation of the mTOR pathway with everolimus and BEZ235 treatment, we examined the expression of pS6 (a downstream target of the mTOR pathway). Both everolimus and BEZ235 caused a reduction in pS6 expression in TCam-2 cells at all the concentrations used (**Figure 3G**). As expected, everolimus did not affect the expression of S6. However, BEZ235 treatment altered S6 expression in TCam-2 cells (**Figure 3G**), which is consistent with a previous observation (35). To understand the effect of everolimus and BEZ235 on other members of the mTOR pathway, cell lysates from treated and control TCam-2 cells were subjected to a mTOR protein array, which allows simultaneous detection of 16 phosphorylated proteins belonging to the mTOR/Akt signaling axis. Treatment with everolimus and BEZ235 reduced the expression level of some of the key components of the mTOR pathway (mTOR Ser2481, S6RP Ser235/236, RSK1 Thr421/Ser424, Bad Ser112 and PRAS40 Thr246; **Figures 3H, I**). The phosphorylation of mTOR at Ser2481 and S6 ribosomal protein at Ser235/236 indicated activation of the mTOR pathway. RSK1 Thr421/Ser424 phosphorylates a wide range of substrates, including ribosomal protein S6, and positively regulates protein translation and cell growth. Bad is a pro-apoptotic protein, and its phosphorylation at Ser112 by Akt promotes cell survival. Phosphorylation of PRAS40 at Thr246 by Akt relieves PRAS40 inhibition of mTORC1 resulting in upregulation of the mTOR pathway. Reduced expression of these components (**Figure 3I**) in the everolimus and BEZ235 treatment groups indicated the suppression of the mTOR pathway among the everolimus and BEZ235 treatment groups.

### Co-treatment of mTOR inhibitors with carboplatin act synergistically against testicular cancer germ cells

**Figure 4:**
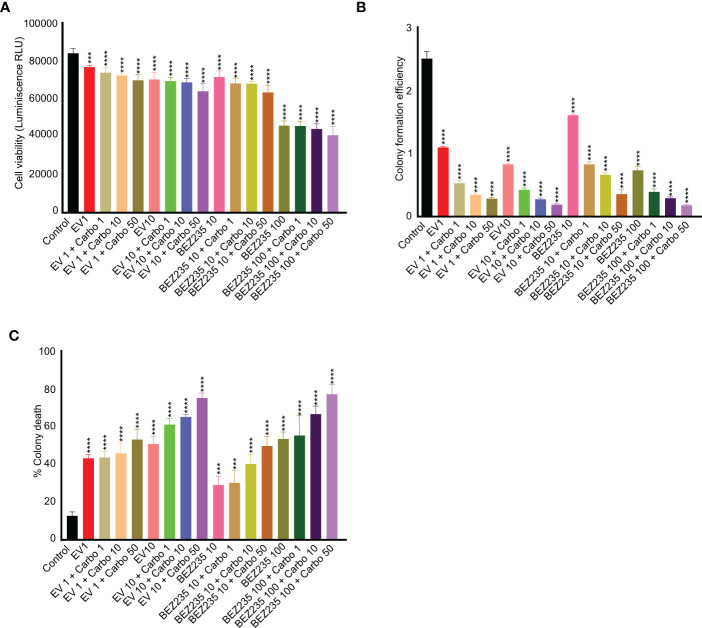
mTOR inhibitors act synergistically with carboplatin in reducing viability and colony formation efficiency of TCam-2 cells. **(A)** TCam-2 cells were cultured in the presence of escalating concentrations of mTOR inhibitors (BEZ-235, 1 and 10 nM, and everolimus, 10 and 100 nM) in combination with carboplatin (1, 10 and 50 ng/ml) for four days. The viability was measured in different treatment groups and is expressed as luminescence (Relative Light Unit, RLU) in comparison to controls (no treatment). **(B, C)** For colony formation assay and colony death, the colonies were counted manually (n = 3; ***P<0.001; ****P<0.0001).

Previous studies have shown that Seminoma patients respond well to carboplatin with 95% relapse-free 5-year survival rate (36). To find if the suppression of the mTOR pathway can synergistically increase the death of cancer cells with chemotherapy drugs, we cultured TCam-2 cells with different concentrations of carboplatin and mTOR inhibitors. A dose-dependent reduction in the viability of TCam-2 cells with increasing concentrations of carboplatin and mTOR inhibitors was observed (**Figure 4A**). Furthermore, the addition of carboplatin reduced the colony formation efficiency and increased the colony death of TCam-2 cells as compared to control and mTOR inhibitor only treatments (**Figures 4B, C**). These results indicate that mTOR inhibitors may potentially improve the outcome of the use of chemotherapy drugs in seminoma patients.

## Discussion

TC is the most common cancer which affects the young population during the phase of high career aspirations and the start of sexual life and can cause infertility and death (37). Despite high sensitivity to chemotherapy, a small fraction of TC patients suffers from relapsed or refractory tumors. In the past 20 years, many targeted treatments have been tried for patients with refractory TC without any success (38–42). With several decades of continuous research efforts, neither the mechanisms involved in resistance development nor promising therapeutic options for patients with relapse of TC have been identified. To this end, we investigated the involvement of LKB1, a tumor suppressor gene, in the development of TC.

TC usually starts as GCNIS and later, alterations in pathways regulating cell fate and proliferation (such as PI3K/Akt/mTOR signaling axis), result in the generation of invasive germ cell tumors. The PI3K/Akt/mTOR signaling axis is well known to play a key role in cancer development and progression. This axis is indispensable for many different functions including, cell proliferation, differentiation, metabolism, cytoskeletal reorganization, and apoptosis (43). A previous study reported activation of the mTOR pathway in 94.4% of patients with intracranial germ cell tumors (44). In the present study, we have shown that *LKB1*, a tumor suppressor gene and negative regulator of the mTOR pathway, is downregulated in TC. LKB1 regulates a broad spectrum of cellular functions, including cell growth, metabolism, autophagy, and polarity by phosphorylation of adenosine monophosphate-activated protein kinase (AMPK) and other AMPK-like kinases (45). With the use of a seminoma cell line (TCam-2), we showed that suppression of the mTOR pathway results in reduced viability and proliferation of cancer germ cells. Inhibition of mTOR with RAD001 in cisplatin-resistant gastric cancer cells has also yielded promising results (46). However, another study has shown only a marginal increase in disease-free survival duration in a fraction of TC patients using everolimus (47). Upstream activation of the mTOR pathway (such as by downregulation of LKB1) could be a reason for poor response to everolimus in patients with loss of PTEN in their study. Moreover, these patients were heavily pre-treated with chemotherapy for TC, which may have resulted in the accumulation of multiple mutations. Therefore, the use of mTOR inhibitors in patients with dysregulated mTOR signaling during the early stage of the disease will potentially provide a better outcome in terms of reduced drug toxicity and relapse of the disease. Moreover, the use of combination therapy needs to be tested on a larger patient cohort.

Patients with the inactivation of the *LKB1* gene are predisposed to early-onset of several different types of tumors (skin, pancreatic, ovarian, lung, and cervical cancers) (19). However, the involvement of LKB1 loss in TC has not yet been reported. We here report the reduced expression of LKB1 in cancerous germ cells. Therefore, targeting the loss-of-function mutation of the *LKB1* gene presents a unique therapeutic approach to treating such cancer patients. In this direction, the establishment of standard genetic and molecular screening tests for *LKB1* gene expression and mutations are critical, which requires costly deep sequencing techniques. To this end, initial screening with immunohistochemical staining for LKB1 expression on cancer tissue samples or biopsies may provide an effective alternative approach. In this study, we used this approach and found decreased expression of LKB1 in human TC patient samples (N=43, 35 seminoma, 8 controls). Another major challenge after detecting LKB1 expression is developing functional assays to measure the activation of LKB1 following treatment with AMPK activators such as phenformin. This can be achieved by the *ex vivo* culture of tumor biopsies. In this direction, we have taken an initial step by using a novel approach of culturing a seminoma cancer cell line (TCam-2) in 3D. Treatment of these cells with BEZ235, a dual inhibitor of mTOR and PI3 kinase, and everolimus, an allosteric mTOR inhibitor, resulted in reduced viability and proliferation of cancer cells. These results provide evidence for the potential use of mTOR inhibitors in TC patients.

## Data availability statement

The original contributions presented in the study are included in the article/supplementary material. Further inquiries can be directed to the corresponding author.

## Ethics statement

The studies involving human participants were reviewed and approved by University of Newcastle Human Ethics Research Committee. The patients/participants provided their written informed consent to participate in this study. The animal study was reviewed and approved by University of Newcastle Animal Care and Ethics Committee.

## Author contributions

MK and PT designed research. MK, SS, and MJ performed the research. MK and PT analysed the data. MK and PT wrote the paper. PT supervised the study, provided financial support, editing and final approval of the manuscript. All authors contributed to the article and approved the submitted version.
